# Salivary TNFα levels in groups of subjects with rheumatoid arthritis and chronic periodontitis

**DOI:** 10.1186/s13104-016-2341-7

**Published:** 2017-01-07

**Authors:** Ehsan B. Gamel, Nada T. Hashim, Asim Satti, Bakri G. Gismalla

**Affiliations:** 1 Faculty of Dentistry, University of Khartoum, Khartoum, Sudan; 2 University of Science and Technology, Omdurman, Sudan

**Keywords:** Periodontitis, Rheumatoid arthritis, TNF-α

## Abstract

**Background:**

Rheumatoid arthritis (RA) and chronic periodontitis are the most common chronic inflammatory diseases with significant pathological and clinical similarities. Numerous studies have indicated a relationship between rheumatoid arthritis and periodontal disease. The aim of this study was to compare the TNF-α levels in saliva among patients with Rheumatoid arthritis (RA) and chronic periodontitis as well as healthy subjects.

**Methods:**

One hundred and seventy-one patients were enrolled in this cross-sectional study. Fifty-seven patients diagnosed of RA, 57 patients with chronic periodontitis and 57 healthy subjects. These patients have been examined with regard to TNF-α level from salivary samples. Their teeth were examined with regard to Plaque Index , Gingival Index, probing depth and clinical attachment level.All patients were non-smokers.

**Results:**

The results revealed a significant difference in all periodontal parameters among the three groups. The chronic periodontitis group showed a significantly higher value in all clinical periodontal parameters in comparison to both the RA and healthy groups. No significant difference was found between salivary TNF-α level among the three study groups.

**Conclusions:**

Patients with chronic periodontitis had the highest periodontal indices. However there was no significant difference regarding the level of salivary TNF-α. Hence, suppression of proinflammatory cytokines might prove beneficial in suppressing periodontal diseases among RA patients.

**Electronic supplementary material:**

The online version of this article (doi:10.1186/s13104-016-2341-7) contains supplementary material, which is available to authorized users.

## Background

Periodontal diseases are destructive inflammatory conditions of the hard and soft tissues surrounding the teeth [1].

Rheumatoid arthritis (RA) is a systemic autoimmune disease that affects 0.5–1.0% of the population, with women carrying a higher risk (3:1) of disease than men [2]. Both RA and periodontitis are characterized by self-maintaining inflammation in a fluid filled compartment adjacent to bone, in which an inflammatory process lead to common clinical symptoms and, ultimately, to destruction of the adjacent bone [3]. In addition, periodontitis has remarkably similar cytokine profiles to RA [4]. As for RA, disease progression seen in chronic periodontitis consists of the continuing presence of high levels of pro-inflammatory cytokines including IL-1β and TNF-α and low levels of anti-inflammatory cytokines like IL-10 and transforming growth factor-beta (TGF-β) [5, 6].

During recent years, there has been increasing evidence suggesting a correlation between RA and periodontitis, a number of well conducted studies have shown that patients with RA have an increased possibility of expressing mild to severe periodontitis compared with healthy people [7, 8].

TNF-α along with several types of inflammatory biomarkers associated with both oral diseases, as well as systemic diseases have been detected in saliva [9].

The study carried out by Abdelsalam et al. in 2010 in Sudan [10] indicated a significant relationship between periodontal disease and RA and highlighted the potential for an association between these chronic inflammatory conditions among the Sudanese population.

Considering these findings, there is a need to further study the factors that may contribute to the relationship between chronic periodontitis and RA. Additionally the presence of common underlying inflammatory pathways mediating the progression of periodontitis and RA would offer potentially important common therapeutic targets [11].

Therefore this research was designed to determine and compare the TNF-α level in saliva among patients with Rheumatoid arthritis (RA), chronic periodontitis, and healthy control subjects.

## Methods

One hundred and seventy-one patients were enrolled in this cross-sectional study. Fifty-seven patients diagnosed of RA as defined by the American College of Rheumatology (ACR) and the European League Against Rheumatism (EULAR) classification, were invited to participate in this study [12].

Fifty-seven patients with chronic periodontitis based on the criteria defined by the American Academy of Periodontology Armitage [13] and 57 healthy subjects were enrolled.

Rheumatoid arthritis subjects were selected from the common rheumatoid arthritis clinics in Khartoum State, while chronic periodontitis and healthy subjects were recruited from the periodontology departments, Faculty of Dentistry—University of Khartoum and Khartoum Dental Teaching Hospital.

Duration of RA as well as drugs used by these patients for the treatment of RA were recorded.

Exclusion criteria included systemic diseases that can affect periodontal status, smoking, antibiotic therapy within the last 3 months and periodontal treatment within the last 6 months. Additionally, subjects having less than 10 teeth were excluded from the study.

### Data collection

Demographic data, duration of the disease since it was diagnosed and type of the anti-rheumatoid drug were taken from records of the patients at the rheumatoid clinics.

### Periodontal examination

A calibrated examiner (periodotist Gamel EB) recorded all clinical periodontal indices; plaque index (PI) [14, 15], gingival index (GI) [16], pocket depth (PD), and CAL for each patient, after the collection of saliva. Measurements for those indices were recorded from six locations per tooth (mesio-buccal, mid-buccal, disto-buccal, mesio-lingual, mid-lingual, and disto-lingual) using Michigan O periodontal probe.

The clinical base line data were attached in a supplementary file.

### Saliva collection

Saliva samples were obtained from all subjects at the day of the intraoral examination. Unstimulated whole saliva was collected in sterile collection tubes from each subject between 9 and 11 a.m. according to the method described by Navazesh [17] as follow:

1. The patient was asked to avoid intake of any food or beverages (except for water) 1 h before the saliva collection.
2. The patient was asked to rinse his/her mouth with tab water and then to take a rest for 5 min.
3. Then the patient was asked to swallow to empty his/her mouth from saliva.
4. After that the patient was asked to sit in an upright position and allow saliva to accumulate in the floor of the mouth and spit it in graduated test tube every 60 s.

Collected samples were put on ice immediately and then frozen at −80 °C until the time of analysis. Forty saliva samples were selected by simple randomization to undergo the ELISA analysis as follow; fifteen samples from the RA group, fifteen from patients with chronic periodontitis and ten samples from the control group. The lottery method was applied for simple randomization. TNF-α was analyzed in salivary samples using Biolegend’s ELISA MAX™ standard set which is a quantitative sandwich Enzyme-Linked Immunosorbent Assay (ELISA). The TNF-α level was expressed in picogram per milliliter (pg/ml).

### Statistical analysis

Statistical Package for the Social Sciences^®^ (SPSS) computer software version 22 was used for analysis of the data obtained. Independent sample’s T test and one way ANOVA were used for comparison of means. Pearson’s correlation was used to correlate the quantitative variables. p value of less than 0.05 considered significant. Confounding variables were statistically controlled. Data was presented in frequencies, tables and graphs (Additional file 1).

## Results

**Fig. 1:**
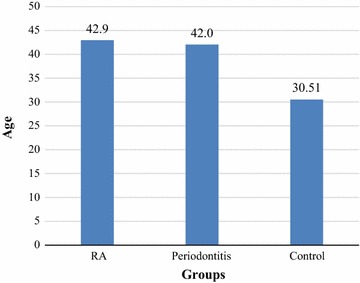
Age means of the RA patients, chronic periodontitis patients and control subjects

**Fig. 2:**
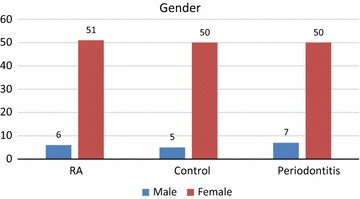
Gender distribution among the RA, chronic periodontitis and control groups

Fifty-seven RA patients together with 57 chronic periodontitis patients and 57 healthy adult subjects participated in the study, with a mean age approximately similar in the RA and chronic periodontitis groups (42.9 ± 10.3) years and (42.0 ± 10.0) years respectively. However, a younger age was found in the control group (30.51 ± 6.00) years (Fig. 1). Subjects were predominantly females in the three groups (Fig. 2).

The medications used by the RA patients were methotrexate, hydroxychloroquine, prednisolone, NSAID and sulphasalazine (Table 1).

**Table 1:** Type of Medication that were used by RA subjects

Drug	Frequency	Percent
Methotrexate	27	21.8
Hydroxychloroquine	44	35.5
Prednisolone	40	32.3
NSAID	5	4
Sulphasalazine	4	3.2

Clinical periodontal measurements were analyzed in two steps: first, the full mouth recordings at six sites/tooth were compared between the study groups; then the levels of salivary TNF-α were compared among the three study groups (Additional file 1).

The mean values of clinical periodontal measurements (PI, GI, PD and CAL) are outlined in Table 2.

**Table 2:** Comparison of the periodontal parameters among the study groups using one way ANOVA test

	RA		Chronic periodontitis		Control		p value
	Mean	SD	Mean	SD	Mean	SD	
PI	0.91	0.32	1.62	0.27	0.23	0.26	0.001
GI	0.99	0.17	1.52	0.33	0.26	0.32	0.001
PDD	0.02	0.04	0.16	0.41	0	0	0.001
CAL	0.51	0.57	0.86	0.77	0	0	0.001

One way ANOVA test performed,  p value is significant

The results revealed a significant difference in all periodontal parameters among the three groups. The periodontal disease group experienced a significantly higher values in all clinical periodontal parameters in comparison to the RA and healthy groups (p value = 0.001) (Table 2).

There was no significant correlation between the type of drug used for treatment of RA and the clinical attachment loss (CAL) (Table 3). Additionally, no significant difference has been obtained when correlating the CAL with the disease duration (p = 0.189).

**Table 3:** The Correlation between the types of the drug used and the Clinical Attachment loss (CAL)

	N	Mean	SD	Sig
Methotrexate	27	0.60	0.68	0.285
Non methotrexate	30	0.43	0.44	
Hydroxychloroquine	44	0.50	0.56	0.832
Non hydroxychloroquine	13	0.54	0.62	
Prednisolone	40	0.54	0.59	0.485
Non prednisolone	17	0.43	0.52	
NSAID	5	0.57	0.51	0.815
Non NSAID	52	0.50	0.57	
Sulphasalazine	4	0.49	0.56	0.953
Non sulphasalazine	53	0.51	0.57	

The mean level of salivary TNF-α levels in the RA patients were(30.80 ± 26.73) pg/ml while in the chronic periodontitis patients and control subjects were (28.70 ± 35.24) pg/ml, (35.98 ± 30.43) pg/ml respectively. When comparing the salivary levels of TNF-α among the 3 study groups using one way ANOVA test, no significant difference was detected (p value = 0.846) (Table 4).

**Table 4:** Comparison of TNF-α salivary level among the study groups

TNF level	N	Mean	SD	95% CI for mean	
				Lower bound	Upper bound
RA	15	30.80	26.73	16.00	45.60
Chronic periodontitis	15	28.70	35.24	9.18	48.22
Control	10	35.98	30.43	14.21	57.74
Total	40	31.31	30.40	21.58	41.03

One way ANOVA performed, p value = 0.846, p value is not significant

No significant correlation was found between the salivary level of TNF-α and CAL in the chronic periodontitis and RA groups (Table 5).

**Table 5:** Correlation between the salivary TNF-α levels and CAL in the RA and chronic periodontitis groups

Group	Correlation	Pearson’s correlation	p value
RA	TNF-CAL	−0.212	0.447
Chronic periodontitis	TNF-CAL	0.38	0.162

p value is not significant

## Discussion

Rheumatoid arthritis (RA) is considered a major representative of a large group of rheumatic diseases whose main manifestation is arthritis [18]. Chronic Periodontitis is a chronic inflammatory disease, characterized by tissue destruction which results from the interaction of microbial dental plaque with host immune defense mechanisms [19].

There are some similarities between the local tissue destruction features of RA and periodontal disease manifested in active tissue destruction of both diseases by the presence of similar cells infiltration as well as inflammatory mediators [20].

In both diseases, TNF-α contributes to the upregulation of osteoclastogenesis and the of osteoblastogenesis [21].

The current study compared the periodontal parameters and the salivary level of TNF-α among RA patients, chronic periodontitis patients and healthy subjects. Additionally it correlated the level of the salivary TNF-α with the CAL in each group.

The results revealed a significant difference in periodontal parameters (PI, GI, PD and CAL) among the RA, chronic periodontitis and control groups, with the highest score detected among the chronic periodontitis group followed by the RA group, while the least score was identified among the control group.

Although increased levels of clinical attachment loss (CAL) may be expected in patients with RA, the long-term usage of anti-rheumatic agents may be responsible for the lesser level of attachment loss among rheumatoid arthritis patient comparing with periodontitis patients, as it has been suggested that treatment of RA with disease modifying anti-rheumatic drugs (DMARD) improves their periodontal condition due to its host modulatory effect, thus masking the gingival inflammation and actual periodontal destruction. This led to reduction in the severity of periodontal diseases among RA group comparing with chronic periodontitis patients [22–24].

This finding is similar to the findings of Mirrielees et al. [25] who found that, the chronic periodontitis group had significantly higher values for all clinical periodontal parameters compared with the RA and healthy groups.

Moreover, when comparing the level of salivary TNF-α among the RA patients, chronic periodontitis patients and controls, the values did not show significant differences, furthermore the amount of clinical attachment loss among the rheumatoid arthritis and chronic periodontitis groups was not correlated with salivary TNF-α level, supporting the idea that levels of inflammatory mediators in periodontitis may be influence by multiple factors such as remission and active period of the disease and local bacterial compositions [26].

Other possibility for the insignificant difference in the level of salivary TNF-α among the three groups and the absence of a correlation between the level of salivary TNF-α among the RA group is that, the DMARD therapy can influence salivary biomarkers of periodontal disease as well as salivary biomarker of RA patients. This reduction of the level of salivary TNF-α could be a reflection of reduced RA disease activity and also might affect the inflammatory components of periodontal disease [25].

This result agrees with the finding of other previous studies [24, 25, 27] however it disagrees with the results obtained by Kobayashi et al. [28] who found a significant difference in the level of serum TNF-α between the RA and control groups.

The finding of the present study highlights prospects to consider biological therapies for periodontitis that would specifically target selected proinflammatory mediators for control in the local oral environment.

## Conclusion

The present data further supports the hypothesis that disregulation of molecular pathways in the inflammatory response causes RA as well as periodontitis.

Immuno-suppressive drugs and NSAIDs decrease the production of inflammatory mediators in RA patients. Consequently without long-term NSAID suppression, RA patients would exhibit higher salivary amounts of these mediators that may possibly lead to increased periodontal destruction [29].

It appears that RA and periodontitis have a significant difference on the clinical periodontal parameters however no significant difference was observe regarding the level of salivary TNF-α.

To understand further the nature and mechanisms of interactions between RA and periodontitis, further longitudinal clinical studies are needed.

## Additional file

**Additional file 1.** Baseline clinical characteristics of the patients.

## Abbreviations

RA: rhumatoid arthritis
TNFα: tumor necrosis factor alpha
PI: plaque index
GI: gingival index
PD: probing depth
CAL: clinical attachment level
ACR: American College of Rheumatology
EULAR: European League Against Rheumatism
TMJ: temporo mandibular joint
DMARD: disease-modifying anti-rheumatic drugs
NSAID: non-steroidal anti-inflammatory drug
